# Socioeconomic Status and Reduced Kidney Function in the Whitehall II Study: Role of Obesity and Metabolic Syndrome

**DOI:** 10.1053/j.ajkd.2011.04.017

**Published:** 2011-09

**Authors:** Talal M. Al-Qaoud, Dorothea Nitsch, Jonathan Wells, Daniel R. Witte, Eric J. Brunner

**Affiliations:** 1 Department of Epidemiology and Public Health, University College London, London, UK; 2 Department of Non-Communicable Disease Epidemiology, Faculty of Epidemiology and Population Health, London School of Hygiene and Tropical Medicine, London, UK; 3 MRC Childhood Nutrition Research Centre, Institute of Child Health, London, UK; 4 Steno Diabetes Center, Gentofte, Denmark

**Keywords:** Reduced kidney function, estimated glomerular filtration rate, lean body mass, obesity, serum lipids, socioeconomic status

## Abstract

**Background:**

Previous US-based studies have found that chronic kidney disease (CKD) disproportionately affects those of more adverse social circumstances. Our aim was to show the association between socioeconomic status (SES) and decreased kidney function in a European context and explore the role of obesity and metabolic syndrome. We consider the potential confounding effect of lean muscle mass.

**Study Design:**

Cross-sectional.

**Setting & Participants:**

White participants in the follow-up of the Whitehall II cohort: UK-based European population (age, 55-79 years; n = 5,533), of whom 4,066 men (73%) and 1,467 women (27%) with complete data were analyzed.

**Predictors:**

Self-reported occupational grade/salary range.

**Outcomes:**

Estimated glomerular filtration rate (GFR) using the CKD-EPI (CKD Epidemiology Collaboration) equation.

**Measurements:**

Body mass index (BMI), serum lipid levels, blood pressure, Tanita TBF-300 body composition analyzer, impedance-derived lean mass index (LMI).

**Results:**

Participants in a lower compared with higher occupational grade were at increased odds of having decreased GFR (age- and sex-adjusted OR, 1.31; 95% CI, 1.12-1.53; *P* = 0.001). Socioeconomic disparity in LMI was evident in women, but not men. After further adjustment for BMI and components of metabolic syndrome, the odds of decreased GFR in whites with a lower compared with higher occupational grade was attenuated by 23.3% (OR, 1.23; 95% CI, 1.06-1.45; *P* = 0.008). Adjustment for LMI explained 15% of the association between SES and estimated GFR.

**Limitations:**

Cross-sectional design, missing data for subset of participants, no urinary data.

**Conclusions:**

BMI and components of metabolic syndrome may explain up to a quarter of the association between low SES and decreased GFR, suggesting potential modifiable factors.

It has been widely documented that those who are less affluent and/or of an ethnic minority background are over-represented in persons who develop established kidney failure.^1-8^ Studies have examined the association of socioeconomic status (SES) at the individual and area levels with prevalence of earlier stages of chronic kidney disease (CKD).^9-18^ Most of these studies have been conducted in the United States on biethnic populations using income and education as measures of SES while simultaneously accounting for the role of multiple behavioral and biological factors, including smoking, alcohol, diabetes, and hypertension.^10-16^

However, the pathways by which this socioeconomic disparity in patients with CKD arises are not fully understood. Metabolic manifestations of obesity (including dyslipidemia, diabetes, and hypertension) have been implicated in the increased cardiovascular risk and progressive renal insult in patients with CKD,^19-23^ with evidence from SHARP (Study of Heart and Renal Protection) and meta-analyses of intervention with statins in dyslipidemic individuals with CKD conferring renal and cardiovascular benefit.^24-27^

Most observational studies have derived data using serum creatinine–based estimated glomerular filtration rate (eGFR). Creatinine is a breakdown product of muscle metabolism; serum creatinine levels are influenced by the muscle mass of the study participant.^28,29^ Similarly, body mass index (BMI), a commonly used proxy of obesity, is influenced by not only fat, but also muscle mass of a person.^19^ Therefore, analyses with outcome creatinine-derived eGFR and exposure BMI as a proxy of obesity may be confounded by muscle mass.

Despite the increasing health burden, the role of both obesity and metabolic syndrome in the SES-CKD association has not been addressed in large population-based cohorts. Analyses to date have not allowed for confounding by socioeconomic differences in muscle mass.

Our aim was to show the prevalence of decreased eGFR according to occupational grade in a large UK-based European population (age, 55-79 years) by conducting a cross-sectional analysis of data from the Whitehall II cohort. We hypothesize that obesity and dyslipidemia have a significant role in explaining the increased risk of decreased eGFR in individuals with a lower compared with higher occupational grade. We include an analysis that adjusts for lean body mass, examining its potential confounding effect in the association between SES, potential risk factors, and eGFR.

## Methods

### Study Population

Data for this cross-sectional analysis were drawn from the Whitehall II cohort study of British civil servants, recruited from men and women working in London government offices in 1985-1988.^30^ The baseline sample consisted of 10,308 participants aged 35-55 years. Phase 9 of the study (2007-2009) was composed of 6,761 multiethnic participants who attended the medical screening and completed the questionnaire, 5,725 of whom were white. Of these eligible participants, a valid creatinine measurement was obtained for 5,533 (96.6%) in addition to data for occupation grade, obesity, and metabolic syndrome components, yielding a sample of 4,066 white men (73%) and 1,467 women (27%) aged 55-79 years at phase 9. The University College London research ethics committee had approved the study protocol, and participants had given informed consent.

### Kidney Function

We used the isotope-dilution mass spectrometry (IDMS)-traceable CKD-EPI (CKD Epidemiology Collaboration) equation,^31^ consistent with the guidelines set by KDOQI (Kidney Disease Outcomes Quality Initiative) and KDIGO (Kidney Disease: Improving Global Outcomes).^32,33^ For white men with a creatinine level <0.9 mg/dL, the formula is 141 × (serum creatinine/0.9)^−0.411^ × (0.993)^age^; for serum creatinine level ≥0.9 mg/dL, it is 141 × (serum creatinine/0.9)^−1.209^ × (0.993)^age^. For white women with a serum creatinine level <0.7 mg/dL, the formula is 144 × (serum creatinine/0.7)^−0.329^ × (0.993)^age^; for serum creatinine level >0.7 mg/dL, it is 144 × (serum creatinine/0.7)^−1.209^ × (0.993)^age^. We analyzed eGFR (expressed in milliliters per minute per 1.73 m^2^) as a continuous variable and a categorical binary variable denoting having or not having decreased GFR (defined here as eGFR <60 mL/min/1.73 m^2^).

### Socioeconomic Position

Occupational grade was self-reported by nonretired participants. For those no longer working in the civil service, information for the most recent occupation grade was filled in from phase 8 back to phase 1. Based on salary (salary ranges shown here are from 1992) and work role, the civil service stratifies a hierarchy of employment grade. Of note, in this article, “higher grade” denotes higher status grade and does not refer to the number assigned to the grade; thus, grade 1 is higher (in occupational status) than grade 7. The highest status grades are considered administrative and comprise unified grades 1-6, with a salary range of £28,904-£87,620 ($53,993-$163,674 at the 1992 conversion factor of 1.868; equivalent to Whitehall II Study grade 1) and unified grade 7, a lower status administrative grade with salary range of £25,330-£36,019 ($47,316-$67,283; Whitehall II grade 2). Executive grades include Senior Executive Officer, salary range of £18,082-£25,554 ($33,777-$47,735; Whitehall II grade 3); Higher Executive Officer, salary range of £14,456-£20,850 ($27,004-$38,948; Whitehall II grade 4); and Executive Officer, salary range of £8,517-£16,668 ($15,910-$31,136; Whitehall II grade 5). Clerical and support staff have a salary range of £7,387-£11,917 ($13,799-$22,261; Whitehall II grade 6).^30^ For the purpose of aiding interpretation and analysis, occupational grade was analyzed in 3 levels of decreasing status: high status, administrative (Whitehall II grades 1-2); medium status, executive (Whitehall II grades 3-5); and low status, clerical and support staff (Whitehall II grade 6).

### Obesity and Components of Metabolic Syndrome

BMI (weight in kilograms divided by height in meters squared) was analyzed in sex-specific quartiles. The Adult Treatment Panel III (ATP III)^34^ defines components of metabolic syndrome as central obesity (waist circumference ≥102 cm in men and ≥88 cm in women), dyslipidemia (triglyceride level ≥150 mg/dL), high-density lipoprotein (HDL) cholesterol level (<40 mg/dL in men and <50 mg/dL in women), blood pressure (≥130/85 mm Hg), and fasting plasma glucose level (≥110 mg/dL). The association of each of these components was assessed independently and simultaneously. Blood pressure was treated as 2 components, systolic and diastolic, for analytic purposes. These measures were used as sex-specific quartiles for stratified analysis and cohort-specific quartiles for pooled analysis.

### Lean Mass Index and Fat- Free Mass

Height was measured in a standard fashion with the head in the Frankfurt plane. The Tanita TBF-300 body composition analyzer (Tanita, www.tanita.com/en/) is a leg-to-leg pressure contact system consisting of 2 subdivided stainless steel footpad electrodes that are mounted on a platform scale, generating impedance of lower extremities.^35^ Fat-free mass (FFM) was calculated directly by the Tanita instrument. A proxy of lean mass index (LMI) was calculated from raw impedance output by the Tanita instrument as described next. The formula used to calculate this proxy of LMI is based on the observation that impedance is a function of total-body water (TBW), such that TBW is proportional to the square of height divided by impedance (R), in other words, TBW ∝ Height^2^/R, which, after adjustment for hydration of lean tissue (HLT), calculates lean mass (LM). Thus, given that LM = TBW/HLT, the relationship can be simplified to LMI is proportional to (Height^2^/R)/HLT/Height^2^, Height^2^ cancels out; thus, LMI ∝ 1/(R × HLT). Our population is of homogenous age, which means that HLT in this formula is a sex-specific constant. When analyses of a population study are conducted adjusted for sex or within each sex, differences in LMI between study participants are driven by the bioimpedance analysis index (BAI) = 1/R, in other words, relative differences in BAI capture relative LMI differences between study participants. BAI as a proxy measure of LMI for population-based research has been validated and used for previous research studies in this area. Full details of the BAI are published elsewhere by Wells et al.^36^

### Statistical Analysis

Descriptive statistics for participants, including crude frequency proportions with χ^2^ tests for trend by occupational grade and crude mean values with age-adjusted tests for trend (using linear regression), are presented stratified by occupational grade and sex. Using age-adjusted linear and logistic regression models, differences in BMI and metabolic syndrome components according to occupation grade and the odds of decreased GFR according to all factors under study were computed stratified by sex as a means of testing the pattern, strength, and magnitude of associations with the main outcome and exposures. Pearson correlation test between LMI, fat-free mass, and waist circumference was calculated. Optimal terms for age adjustment were tested using log-likelihood ratio test, with no evidence of nonlinearity of the association (likelihood ratio test, *P* = 0.9 for men and *P* = 0.7 for women [quadratic model] and *P =* 0.9 for men and *P =* 0.9 for women [categorical model]).

Multivariable logistic regression models were computed to determine the presence and magnitude of the association between low SES and decreased GFR using occupational grade. Log-likelihood ratio tests were used to test for a sex–occupational grade interaction. The potential significant mediating role of obesity and metabolic syndrome components was explored by adding each factor independently to the multivariable logistic model containing age, sex, occupational grade, and decreased GFR and calculating the percentage of attenuation by each factor, independently and simultaneously, by subtracting the log odds of decreased GFR after adding factors to the model from the log odds of decreased GFR of the basic model, then dividing by the log odds of decreased GFR of the basic model. In a subgroup of 4,838 participants with impedance measurements (3,627 men and 1,211 women), the confounding role of muscle mass in the associations between decreased GFR and all factors under study was addressed by adjusting for the proxy of LMI (ie, bioimpedance analysis index).

Sensitivity analyses were carried out using eGFR calculated using the IDMS-traceable MDRD (Modification of Diet in Renal Disease) Study equation^37^ and also using linear regression analyses with comparable results that were consistent with those shown in this report. Adjustment for prevalent diabetes and non-HDL cholesterol level also was conducted. Statistical analyses were conducted using Stata, version 10 (StataCorp LP, www.stata.com).^38^

## Results

### Participant Characteristics

**Figure 1:**
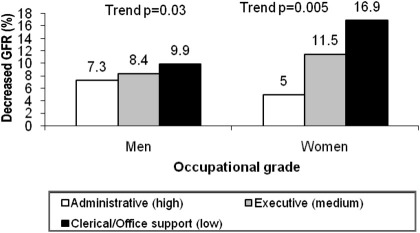
Percentage of participants with decreased glomerular filtration rate (GFR) by occupational grade and sex. *P* value for trend calculated using a regression model adjusted for age. Decreased GFR defined as GFR estimate <60 mL/min/1.73 m^2^ using the isotope-dilution mass spectrometry–traceable CKD-EPI (Chronic Kidney Disease Epidemiology Collaboration) equation. Total percentage of cases was 7.8% in men and 11.0% in women.

Of 5,533 white European participants (4,066 men and 1,467 women), men had a mean age of 65.5 years, and women, 65.9 years. Most men were in the top administrative grade (58.6%) and nearly half the women were in the middle executive grade (49.7%). Age-adjusted mean eGFRs were similar in men and women (79.7 and 79.4 mL/min/1.73 m^2^). Prevalences of eGFR <60 mL/min/1.73 m^2^ in the cohort were 7.8% for men and 11.0% for women (Fig 1). The highest percentage of obesity was observed in men and women of clerical/office support compared with higher occupational grades (*P* < 0.001; Table 1). However, men of all occupational grades had a less favorable level of each component of metabolic syndrome compared with women of all occupational grades (Table 1). Lower occupational grade was associated with a higher prevalence of decreased GFR compared with a higher occupational grade in both sexes, and women of clerical/office support grade had a lower percentage of eGFR category >90 mL/min/1.73 m^2^ compared to men of clerical/office support grade (Fig 1; Table 2).

### Association of Age, Obesity, and Metabolic Syndrome Components With Decreased GFR

A 1-year increase in age conferred a 15%-16% increased odds of decreased GFR. Using sex-specific quartiles of variables (Table 3), obese men and women compared with underweight/normal-weight men and women had 96% and 101% increased odds of decreased GFR after adjustment for age, respectively. Of metabolic syndrome components, increasing triglyceride levels are linked most clearly to GFR, with individuals in the highest compared with the lowest quartile having 177% and 155% increased odds of decreased GFR for men and women, respectively. Having diabetes was associated with increased odds of decreased GFR in men, but not women. Associations of factors with kidney function as a continuous measure are listed in Table S1 (available as online supplementary material).

### Fat-Free Mass and LMI

In participants with body impedance measures, socioeconomic disparity in raw impedance and differences in LMI was evident in women, but not men (*P =* 0.01 and *P* = 0.3 for impedance; *P =* 0.01 and *P =* 0.2 for LMI, respectively). In both men and women, there was an association between decreased eGFR and increasing quartiles of LMI (*P* < 0.001 for both; data not shown). After adjustment for LMI, the negative association of being in the highest versus lowest quartile of triglyceride levels with GFR remains in both sexes, and the protective association of HDL cholesterol level remains in men only (Tables S2 and S3). Additional LMI adjustment attenuates associations of the remaining components of metabolic syndrome to insignificance in men and all components except BMI in women (Table S3). Adjustment for fat-free mass generated by the Tanita instrument as a measure of muscle mass showed similar results (data not shown). LMI and fat-free mass correlated highly with waist circumference in men and women (Pearson coefficient of 0.4 in men and 0.5 in women for LMI and 0.6 for men and 0.7 for women for fat-free mass).

### Occupational Gradient in CKD and the Role of Obesity and Metabolic Syndrome

Participants with a lower occupational grade were at 31% increased odds of decreased GFR (age- and sex-adjusted odds ratio [OR], 1.31; 95% confidence interval [CI], 1.12-1.53; *P* = 0.001) in comparison to those in a higher occupational grade (Table 4). There was no evidence for a difference in the occupational grade effect on GFR between men and women (Table 4; *P* = 0.3 for likelihood ratio test). Adjusting for BMI attenuated the association by 14.4%, and adjustment for all components of metabolic syndrome attenuated the association by 20%. Adjustment for serum HDL cholesterol and triglyceride (dyslipidemia) levels decreased the excess odds of having decreased GFR in individuals in lower grades by 17.6%, whereas adjustment for BMI and dyslipidemia combined decreased the risk by 23.3%. Adjustment for non-HDL cholesterol level and prevalent diabetes (data not shown) did not attenuate the odds appreciably. Adjustment for age, sex, BMI, and all components of metabolic syndrome did not result in greater attenuation. The occupation grade effect remained significant in the maximally adjusted model.

In the sample with impedance measurements, participants in lower occupation grades were at 13% increased odds of decreased GFR, and analysis stratified by sex shows that the effect was significant in women only (Table S2), with women in lower occupational grades having a 55% increased odds of decreased GFR compared with those in higher occupation grades (*P* = 0.02; *P* = 0.09 for likelihood ratio test for sex–SES grade interaction). Adjustment for LMI explained 15% of the SES-GFR association in the sample (adjusted OR, 1.11; 95% CI, 0.90-1.37) and 6% of the association in women (adjusted OR, 1.51; 95% CI, 1.03-2.23).

## Discussion

We show in a UK white population of 5,533 men and women that individuals of lower SES more often have early kidney function decrease compared with those of higher status measured using civil service occupational grade. Our study implicates obesity and metabolic syndrome in the association between SES and decreased GFR, whereby each independently explains about one-sixth and in combination account for almost a quarter of the increased odds of having decreased GFR seen in individuals with lower status.

Our results are in line with previous studies that have examined the role of individual income and education in CKD.^10,11,13,14^ In contrast to our findings, Peralta et al^16^ found that occupation (based on white- and blue-collar criteria) was not associated with kidney function in either African American or white participants from the Cardiovascular Health Study. Using a life course approach, Shoham et al^15^ showed that ORs for CKD in working class versus non–working class individuals at age 30 years were 1.4 (95% CI, 1.0-1.9) in whites and 1.8 (95% CI, 1.1-3.0) in blacks. A case-control study in Sweden^17^ involving 1,894 participants aged 18-74 years used highest occupation grade of spouses and parents as a measure of household SES and found that women in families with only unskilled workers had a 2-fold increase in odds of CKD in comparison to women in families of professionals. The corresponding excess odds of preuremic CKD in men was 60% in the lowest versus highest household SES. These findings show that associations between grade/social class and CKD are present for both men and women. None of the studies mentioned to date explored the role of dyslipidemia on the occupational gradient of CKD, but most studies addressed the role of BMI, hypertension, and diabetes. In addition, a study that has attempted to explore the role of lipid levels and other factors associated with metabolic syndrome carried out by Kurella et al^20^ showed that in 10,096 nondiabetic individuals with normal baseline kidney function from the ARIC (Atherosclerosis Risk in Communities) trial, participants with metabolic syndrome (ATP III guidelines) had an adjusted 43% increased odds of developing CKD after a 9-year follow-up (OR for eGFR <60 mL/min/1.73 m^2^ using the MDRD Study equation, 1.43; 95% CI, 1.18-1.73). For individual metabolic syndrome traits, except for impaired fasting glucose level, each component of metabolic syndrome was associated with a significantly increased risk of CKD, a finding similar to our study's results. Individuals with increased triglyceride and low HDL cholesterol levels were at 34% and 27% increased odds of developing CKD. Even after adjustment for the subsequent development of diabetes and hypertension during the 9-year follow-up, the OR of incident CKD remained significant, emphasizing that individuals with metabolic syndrome have an independent increased risk of incident CKD. Diabetes accounted for 3% of the SES-GFR association in our sample. However, this should be interpreted with caution because our approach was cross-sectional and the well-established longitudinal association between diabetes and CKD would have been examined better using data for actual date of diagnosis. The obscured effect of systolic blood pressure on kidney function also may have been underestimated because of measurement error.

Our adjustment for LMI as a proxy measure of muscle mass shows how sex differences in muscle mass influence creatinine-based equations. Adjustment for LMI explained 15% of the SES-GFR association in the sample as a whole, suggesting that LMI is a positive confounder of the SES-GFR association, as hypothesized. Despite the absence of data for direct measurement of GFR in our study for comparison, these results point to the importance of adjustment for lean body mass or muscle mass in creatinine-based eGFR.

Strengths of this study include use of a large cohort, use of occupation grade as an individual's measure of SES in a stratified sample of working environment within the UK civil service independent of racial and ethnic effects, adjustments for proxies of lean body mass, and use of the recently devised CKD-EPI equation to estimate GFR.^31^ Our study is novel in terms of addressing the role of obesity and all factors of metabolic syndrome simultaneously and in our exploration of the confounding role of muscle mass in the associations between SES, risk factors, and eGFR.

However, our study does not come without limitations, and caution has to be taken when interpreting our findings. Limitations inherent in cross-sectional studies operate here. Of major importance is our inability to ascertain firm causality between SES and decreased GFR and the potential mediating role of obesity and metabolic syndrome. Another main limitation is the absence of urinary markers (proteinuria/albuminuria). Relating to our measure of SES, occupational grade was based on salary and work role, pointing to the limitation that salary may not reflect SES, depending on nonwage income and number of wage earners in the family unit. Although these factors potentially are important for women in our study, we observe clearer SES differences in decreased GFR prevalence in women than men. Additionally, there is overlap in income at the margins of the salary ranges for each grade. However, income is only one of several defining features of SES, and occupational grade in the civil service is a strong predictor of health outcomes, health behaviors, and aging outcomes.^39-41^ Regarding demographic features of our sample from the Whitehall II cohort, the small number of men in the lowest occupational grade may have limited the power of the analysis to detect differences, and Fig 1 suggests a shallower gradient in men than women between the middle and top occupational grades. Apart from the usual problem of not having gold-standard measurements of GFR (outcome measurement error), there might be additional measurement bias as a result of our use of the Tanita instrument to obtain BMI and impedance to estimate LMI in our adjusted analysis. However, our use of LMI over fat-free mass output from the Tanita instrument previously has been shown to be a more accurate method to quantify total-body water.^36^ Losses to follow-up are inevitable in longitudinal cohorts, and our participants were gathered from a sample representing 67% of the original cohort size (10,308). Our analyses of a sample of whites might be regarded as a limitation given the clear racial disparity in end-stage renal disease. However, our design (restricted to whites) ensured that there was no confounding by ethnic minority status.

Obesity and dyslipidemia are potentially plausible factors that may explain the socioeconomic disparity in early kidney function decline.

## Figures and Tables

**Table 1:** Characteristics of Study Participants by Socioeconomic Status (Occupational Grade)

Characteristic	Men					Women				
	Total (n = 4,066)	Clerical/Office Support	Executive	Administrative	*P* for Trend	Total (n = 1,467)	Clerical/Office Support	Executive	Administrative	*P* for Trend
Total	4,066 (73)	141 (3.5)	1,539 (37.9)	2,386 (58.6)		1,467 (27)	338 (23.0)	729 (49.7)	400 (27.3)	
Age (y)	65.5 ± 5.8	65.5 ± 5.9	65.1 ± 5.8	65.8 ± 5.8	<0.001	65.9 ± 6.1	68.3 ± 6.0	65.7 ± 6.0	64.0 ± 5.6	<0.001
Age group					0.007					<0.001
55-59 y	822 (20.2)	31 (21.9)	354 (23.0)	437 (18.3)		301 (20.5)	34 (10.0)	146 (20.0)	121 (30.3)	
60-64 y	1,339 (32.9)	43 (30.5)	522 (33.9)	774 (32.4)		427 (29.1)	81 (23.9)	218 (29.9)	128 (32.0)	
65-69 y	833 (20.5)	27 (19.2)	289 (18.8)	517 (21.7)		299 (20.4)	67 (19.8)	151 (20.7)	81 (20.2)	
70-74 y	752 (18.5)	31 (21.9)	267 (17.3)	454 (19.0)		317 (21.6)	106 (31.3)	160 (22.0)	51 (12.8)	
≥75 y	320 (7.9)	9 (6.4)	107 (6.9)	204 (8.6)		123 (8.4)	50 (14.8)	54 (7.4)	19 (4.7)	
BMI (kg/m^2^)	26.5 ± 3.9	27.1 ± 4.7	26.8 ± 4.0	26.4 ± 3.8	0.001^a^	27.1 ± 5.5	27.9 ± 5.4	27.2 ± 5.5	25.9 ± 5.4	<0.001^a^
BMI category					0.001					<0.001
Underweight/normal (<18.5-<25 kg/m^2^)	1,482 (36.5)	50 (35.5)	518 (33.7)	914 (38.3)		604 (41.2)	111 (32.8)	281 (38.5)	212 (53.0)	
Overweight (25-<30 kg/m^2^)	1,920 (47.3)	55 (39.0)	752 (48.8)	1,113 (46.7)		474 (32.3)	116 (34.4)	257 (35.3)	101 (25.2)	
Obese (≥30 kg/m^2^)	664 (16.2)	36 (25.5)	269 (17.5)	359 (15.0)		389 (26.5)	111 (32.8)	191 (26.2)	87 (21.8)	
Waist circumference (cm)	95.3 ± 10.8	96.5 ± 13.6	95.9 ± 11.1	94.9 ± 10.5	0.001^a^	85.2 ± 12.8	87.4 ± 12.8	85.0 ± 12.4	83.0 ± 13.2	<0.001^a^
Systolic BP (mm Hg)	126.0 ± 15.4	121.8 ± 14.7	126.2 ± 15.4	126.2 ± 15.5	0.002^a^	123.2 ± 17.4	124.9 ± 16.4	124.4 ± 18.3	119.6 ± 16.2	0.06^a^
Diastolic BP (mm Hg)	71.8 ± 10.1	89.2 ± 9.6	72.0 ± 10.1	71.7 ± 10.0	0.2^a^	69.4 ± 10.2	69.8 ± 10.2	69.7 ± 10.2	68.8 ± 10.2	0.2^a^
HDL cholesterol (mg/dL)	58.3 ± 15.4	55.4 ± 15.4	57.4 ± 15.2	59.0 ± 15.6	<0.001^a^	72.3 ± 18.6	69.1 ± 17.2	71.3 ± 17.9	76.9 ± 20.4	<0.001^a^
Triglycerides (mg/dL)	114.4 ± 70.3	123.1 ± 30.5	118.0 ± 28.8	111.6 ± 29.0	0.006^a^	103.7 ± 54.8	111.7 ± 27.2	105.6 ± 26.3	93.6 ± 24.7	<0.001^a^
Fasting glucose (mg/dL)	96.9 ± 22.0	96.1 ± 22.5	98.2 ± 25.1	96.2 ± 19.7	0.03^a^	93.9 ± 22.7	95.3 ± 27.2	94.1 ± 22.6	92.5 ± 18.5	0.3^a^
Diabetes prevalence					0.002					0.01
No	3,652 (89.8)	120 (85.1)	1,358 (88.2)	2,174 (91.1)		1,302 (88.8)	292 (86.4)	639 (87.6)	371 (92.8)	
Yes	414 (10.2)	21 (14.9)	181 (11.8)	212 (8.9)		165 (11.2)	46 (13.6)	90 (12.4)	29 (7.2)	

*Note:* Categorical values shown as number (percentage); continuous variables, as mean ± standard deviation by occupational grade in men and women. For purposes of the analysis in this article, clerical and office support is considered low occupational grade; executive, medium grade; and administrative, high grade. Conversion factors for units: HDL cholesterol in mg/dL to mmol/L, ×0.02586; triglycerides in mg/dL to mmol/L, ×0.01129; glucose in mg/dL to mmol/L, ×0.05551. Abbreviations: BMI, body mass index; BP, blood pressure; HDL, high density lipoprotein.

a Age-adjusted *P* values for trend.

**Table 2:** Distribution of Men and Women Across eGFR Categories by Socioeconomic Status (Occupational Grade)

eGFR Category^a^	Men					Women				
	Total (n = 4,066)	Clerical/Office Support	Executive	Administrative	*P* for Trend	Total (n = 1,467)	Clerical/Office Support	Executive	Administrative	*P* for Trend
					0.3^b^					<0.001^b^
≥90	1,035 (25.5)	44 (31.2)	406 (26.4)	585 (24.5)		419 (28.6)	72 (21.3)	210 (28.8)	137 (34.3)	
60-89	2,713 (66.7)	83 (58.9)	1,004 (65.2)	1,626 (68.2)		887 (60.5)	209 (61.8)	435 (59.7)	243 (60.8)	
45-59	262 (6.4)	12 (8.5)	103 (6.6)	147 (6.2)		127 (8.7)	47 (13.9)	62 (8.5)	18 (4.5)	
30-44	47 (1.2)	2 (1.4)	21 (1.4)	24 (1.0)		30 (2.0)	9 (2.7)	20 (2.7)	1 (0.3)	
<30	9 (0.2)	0 (0)	5 (0.3)	4 (0.1)		4 (0.3)	1 (0.3)	2 (0.3)	1 (0.2)	

*Note:* Values shown are number (percentage). For purposes of the analysis in this article, clerical and office support is considered low occupational grade; executive, medium grade; and administrative, high grade. Abbreviation: eGFR, estimated glomerular filtration rate.

a eGFR calculated using the CKD-EPI (Chronic Kidney Disease Epidemiology Collaboration) equation and given in mL/min/1.73 m^2^.

b Unadjusted *P* value for trend.

**Table 3:** Association of Decreased GFR With BMI and Metabolic Syndrome Components

Factor	Men (n = 4,066)		Women (n = 1,467)	
	OR (95% CI)	*P*	OR (95%CI)	*P*
Age (/1-y increase)	1.16 (1.14-1.18)	<0.001	1.15 (1.12-1.18)	<0.001
Occupational grade (/grade decrease)	1.26 (1.03-1.54)	0.03	1.43 (1.11-1.83)	0.005
BMI^a^				
Overweight	1.66 (1.26-2.19)	<0.001	1.56 (1.02-2.40)	0.03
Obese	1.96 (1.38-2.80)	<0.001	2.01 (1.31-3.09)	0.001
Waist circumference^b^				
Q2	1.52 (1.04-2.21)	0.02	1.19 (0.70-2.03)	0.5
Q3	1.82 (1.27-2.62)	0.001	1.37 (0.81-2.31)	0.2
Q4	2.02 (1.41-2.90)	<0.001	1.68 (1.01-2.80)	0.04
Systolic BP^b^				
Q2	0.64 (0.45-0.91)	0.02	1.00 (0.57-1.75)	0.9
Q3	0.71 (0.50-1.01)	0.06	1.30 (0.77-2.19)	0.3
Q4	1.18 (0.87-1.61)	0.3	1.42 (0.85-2.39)	0.2
Diastolic BP^b^				
Q2	0.80 (0.57-1.14)	0.2	1.14 (0.69-1.90)	0.6
Q3	0.94 (0.67-1.30)	0.7	1.00 (0.59-1.69)	0.9
Q4	1.42 (1.03-1.95)	0.03	1.93 (1.21-3.09)	0.005
HDL cholesterol^b^				
Q2	0.68 (0.50-0.92)	0.01	0.63 (0.40-1.00)	0.05
Q3	0.66 (0.46-0.95)	0.03	0.65 (0.41-1.03)	0.06
Q4	0.50 (0.36-0.71)	<0.001	0.65 (0.39-1.07)	0.08
Triglycerides^b^				
Q2	1.57 (1.10-2.25)	0.01	1.35 (0.75-2.43)	0.3
Q3	2.14 (1.53-3.00)	<0.001	2.53 (1.56-4.11)	<0.001
Q4	2.77 (1.93-3.97)	<0.001	2.55 (1.57-4.13)	<0.001
Fasting glucose^b^				
Q2	0.86 (0.61-1.22)	0.4	0.73 (0.44-1.20)	0.2
Q3	0.92 (0.65-1.29)	0.6	1.09 (0.68-1.76)	0.7
Q4	1.14 (0.83-1.57)	0.4	1.46 (0.93-2.29)	0.09
Diabetes prevalence (yes vs no)	1.54 (1.11-2.14)	0.009	1.02 (0.62-1.66)	0.9

*Note:* ORs shown are age adjusted by sex for the association of decreased GFR (eGFR <60 mL/min/1.73 m^2^ according to the IDMS-traceable CKD-EPI equation) with BMI and components of metabolic syndrome (each row refers to a separate age-adjusted model). Conversion factors for units: HDL cholesterol in mg/dL to mmol/L, ×0.02586; triglycerides in mg/dL to mmol/L, ×0.01129; glucose in mg/dL to mmol/L, ×0.05551. Abbreviations: BMI, body mass index; BP, blood pressure; CI, confidence interval; CKD-EPI, Chronic Kidney Disease Epidemiology Collaboration; eGFR, estimated GFR; GFR, glomerular filtration rate; HDL, high-density lipoprotein; IDMS, isotope-dilution mass spectrometry; OR, odds ratio; Q, quartile**.**

a Reference group is participants in underweight/normal-weight category.

b Reference group is lowest quartile (Q1) for each set of sex-specific quartiles of factors.

**Table 4:** Association of Decreased GFR With Lower Versus Higher Occupational Grade

Adjustment	Factor	OR (95% CI)	*P*	% Attenuation	*P* for Sex Interaction	LRT *P*^a^
Base model^b^	+ Age, sex	1.31 (1.12-1.53)	0.001	—	0.3	Age, <0.001; sex, 0.2
Obesity model	+ BMI	1.26 (1.08-1.47)	0.004	14.4	0.4	<0.001
Components of MS model	+ Waist circumference	1.28 (1.09-1.49)	0.002	8.6	0.3	<0.001
	+ Systolic BP	1.31 (1.13-1.54)	0.001	0	0.3	0.07
	+ Diastolic BP	1.32 (1.13-1.54)	0.001	0	0.3	0.003
	+ HDL cholesterol	1.27 (1.09-1.49)	0.003	11.1	0.4	<0.001
	+ Triglycerides	1.25 (1.07-1.47)	0.005	17.6	0.4	<0.001
	+ Fasting glucose	1.31 (1.12-1.53)	0.001	0	0.2	0.07
	+ All MS components	1.24 (1.07-1.46)	0.001	20	0.4	<0.001
Dyslipidemia model	+ HDL cholesterol, triglycerides	1.25 (1.09-1.49)	0.006	17.6	0.4	<0.001
Obesity & dyslipidemia model	+ BMI, HDL cholesterol, triglycerides	1.23 (1.05-1.45)	0.009	23.3	0.5	<0.001
Obesity & MS	All factors combined	1.23 (1.06-1.45)	0.008	23.3	0.5	<0.001

*Note:* ORs shown are of decreased GFR (eGFR <60 mL/min/1.73 m^2^ according to the IDMS-traceable CKD-EPI equation) in lower versus higher occupational grades adjusted for age, sex, and further sequential adjustments for obesity (using BMI) and MS components (each model includes basic model in addition to specified factors). N = 5,533. Abbreviations: BMI, body mass index; BP, blood pressure; CI, confidence interval; CKD-EPI, Chronic Kidney Disease Epidemiology Collaboration; eGFR, estimated GFR; GFR, glomerular filtration rate; HDL, high-density lipoprotein; IDMS, isotope-dilution mass spectrometry; LRT, likelihood ratio test; MS, metabolic syndrome; OR, odds ratio**.**

a LRT *P* value indicates the significance of adding variables to the model.

b Test for sex difference in grade effect in CKD, *P* = 0.3 (LRT).

## Supplementary Materials

Supplementary Table S1 (PDF) Age-adjusted association of BMI and components of metabolic syndrome with eGFR as a continuous measure, stratified by sex.

Supplementary Table S2 (PDF) Age-adjusted association of decreased GFR with BMI and components of the metabolic syndrome, by sex, in participants with impedance measurements.

Supplementary Table S3 (PDF) Age- and lean mass index–adjusted association of decreased GFR with BMI and components of the metabolic syndrome, by sex, in participants with impedance measurements.
