# Supplementary material for: Socioeconomic Status and Reduced Kidney Function in the Whitehall II Study: Role of Obesity and Metabolic Syndrome
Source: Am J Kidney Dis. 2011 Sep;58(3):389–97. doi: 10.1053/j.ajkd.2011.04.017 (PMC3192873; doi:10.1053/j.ajkd.2011.04.017)
Supplement: Supplementary Table S1 (PDF) — Age-adjusted association of BMI and components of metabolic syndrome with eGFR as a continuous measure, stratified by sex. [file mmc1.pdf]

Table S1. Age adjusted association of BMI and components of MS with eGFR as a continuous measure (derived from IDMS calibrated CKD-EPI formula, in ml/min/1.73m^2) stratified by sex (each row refers to a separate model on the same set of participants)

	**Men (N=4066)**		**Women (N=1467)**	
**Factor**	**Beta-coefficient(95%CI)**	**p-value**	**Beta-coefficient(95%CI)**	**p-value**
Age* (years)	-5.38(-5.75 to -4.99)	<0.001	-6.12(-6.78 to -5.45)	<0.001
Occupational grade (per grade increase)	-0.42(-1.09 to 0.25)	0.2	-0.99(-1.99 to 0.003)	0.05
BMI* (weight(kg)/height(m^2))	-1.48(-1.91 to -1.06)	<0.001	-1.22(-1.77 to -0.66)	<0.001
Waist circumference* (cm)	-1.42(-1.84 to -0.99)	<0.001	-1.15(-1.79 to -0.49)	<0.001
Systolic BP* (mmHg)	-0.41(-0.80 to -0.01)	0.04	-0.41(-1.07 to 0.23)	0.2
Diastolic BP* (mmHg)	-0.73(-1.11 to -0.35)	<0.001	-0.72(-1.40 to -0.04)	0.04
HDL cholesterol* (mg/dL)	1.70(1.28 to 2.12)	<0.001	0.71(0.07 to 1.35)	0.03
Triglycerides* (mg/dL)	-1.08(-1.44 to -0.73)	<0.001	-2.26(-3.09 to -1.43)	<0.001
Fasting glucose* (mg/dL)	-0.25(-0.65 to 0.15)	0.2	-0.60(-1.31 to 0.10)	0.09
Diabetes prevalence (yes vs. no)	-0.42(-1.67 to 0.81)	0.5	1.16(-1.02 to -3.35)	0.3

* Beta- coefficient represents change in eGFR in mL/min/1.73m^2 per one standard deviation increase in the factor
-Abbreviations: BMI: body mass index, BP: blood pressure, eGFR: estimated glomerular filtration rate, HDL: high density lipoprotein cholesterol. . -Conversion factors: HDL cholesterol mg/dL to mmol/L, x 0.02586; Triglycerides mg/dL to mmol/L, x 0.01129; Fasting glucose mg/dL to mmol/L, x 0.05551. -LRT test for inclusion of quadratic term was significant in men and women (p=0.01, p=0.04)
