# Supplementary material for: Socioeconomic Status and Reduced Kidney Function in the Whitehall II Study: Role of Obesity and Metabolic Syndrome
Source: Am J Kidney Dis. 2011 Sep;58(3):389–97. doi: 10.1053/j.ajkd.2011.04.017 (PMC3192873; doi:10.1053/j.ajkd.2011.04.017)
Supplement: Supplementary Table S2 (PDF) — Age-adjusted association of decreased GFR with BMI and components of the metabolic syndrome, by sex, in participants with impedance measurements. [file mmc2.pdf]

Table S2. Age adjusted odds ratios (with their 95% confidence intervals (CI)) by sex for the associations between CKD stages 3-5 (eGFR <60 ml/min/1.73m^2 derived from IDMS calibrated CKD-EPI formula) and body-mass index (BMI), and components of the metabolic syndrome amongst sample with impedance measurements (each row refers to a separate model on the same set of participants)

Factor	Men (N=3627) Odds ratio(95%CI)	p-value	Women (N=1211) Odds ratio(95%CI)	p-value
Age (per 1 year increase)	1.17(1.14 to 1.20)	<0.001	1.12(1.07 to 1.18)	<0.001
Occupational grade (per grade increase)	1.00(0.78 to 1.29)	0.9	1.55(1.06 to 2.28)	0.02
BMI** (weight(kg)/height(m^2))				
Overweight	1.76(1.27 to 2.44)	0.001	2.11(1.08 to 4.11)	0,02
Obese	1.77(1.13 to 2.76)	0.01	2.57(1.29 to 5.13)	0,007
Waist circumference* (cm)				
Q2	1.68(1.08 to 2.61)	0.02	1.24(0.57 to 2.67)	0.6
Q3	1.92(1.24 to 2.95)	0.003	1.01(0.44 to 2.27)	0.9
Q4	1.87(1.19 to 2.91)	0.006	1.55(0.72 to 3.32)	0.3
Systolic BP* (mmHg)				
Q2	0.62(0.41 to 0.93)	0.03	0.55(0.26 to 1.17)	0.1
Q3	0.63(0.42 to 0.94)	0.02	0.71(0.36 to 1.41)	0.3
Q4	1.01(0.70 to 1.46)	0.9	0.38(0.16 to 0.89)	0.03
Diastolic BP* (mmHg)				
Q2	0.74(0.49 to 1.10)	0.2	0.52(0.26 to 1.03)	0.06
Q3	0.88(0.60 to 1.30)	0.5	0.33(0.14 to 0.78)	0.01
Q4	1.23(0.83 to 1.80)	0.3	0.74(0.38 to 1.45)	0.4
HDL cholesterol* (mg/dL)				
Q2	0.59(0.41 to 0.84)	0.004	0.65(0.32 to 1.29)	0.2
Q3	0.52(0.33 to 0.81)	0.004	0.57(0.28 to 1.16)	0.1
Q4	0.42(0.28 to 0.63)	<0.001	0.49(0.22 to 1.11)	0.09
Triglycerides* (mg/dL)				
Q2	1.58(1.04 to 2.40)	0.03	0.85(0.34 to 2.14)	0.7
Q3	2.09(1.40 to 3.10)	<0.001	2.16(1.07 to 4.35)	0.03
Q4	2.67(1.73 to 4.11)	<0.001	1.97(0.96 to 4.03)	0.06
Fasting glucose* (mg/dL)				
Q2	1.08(0.72 to 1.62)	0.7	0.75(0.37 to 1.48)	0.4
Q3	0.99(0.66 to 1.50)	0.9	0.68(0.32 to 1.44)	0.3
Q4	1.19(0.81 to 1.77)	0.4	0.77(0.37 to 1.61)	0.5
Diabetes prevalence (yes vs. no)	1.84(1.27 to 2.60)	0.001	0.81(0.35 to 1.85)	0.6

*Sex specific quartiles of factors, OR demonstrates comparison to lowest quartile (Q1), **BMI OR demonstrates comparison to participants in underweight/normal category -Abbreviations: BMI: body mass index, BP: blood pressure, HDL: high density lipoprotein cholesterol. -Conversion factors: HDL cholesterol mg/dL to mmol/L, x 0.02586; Triglycerides mg/dL to mmol/L, x 0.01129; Fasting glucose mg/dL to mmol/L, x 0.05551
