# Supplementary material for: Socioeconomic Status and Reduced Kidney Function in the Whitehall II Study: Role of Obesity and Metabolic Syndrome
Source: Am J Kidney Dis. 2011 Sep;58(3):389–97. doi: 10.1053/j.ajkd.2011.04.017 (PMC3192873; doi:10.1053/j.ajkd.2011.04.017)
Supplement: Supplementary Table S3 (PDF) — Age- and lean mass index–adjusted association of decreased GFR with BMI and components of the metabolic syndrome, by sex, in participants with impedance measurements. [file mmc3.pdf]

Table S3. Age and lean mass index (LMI) adjusted odds ratios (95% confidence intervals (CI)) by sex for the associations between decreased GFR (eGFR <60 ml/min/1.73m^2 derived from IDMS calibrated CKD-EPI formula) and body-mass index (BMI), and components of the metabolic syndrome amongst sample with impedance measurements (each row refers to a separate model on the same set of participants)

Factor	Men (N=3627) Odds ratio (95%CI)	p-value	Women (N=1211) Odds ratio (95%CI)	p-value
Occupational grade (per grade increase)	0.99 (0.77 to 1.29)	0.9	1.51 (1.03 to 2.23)	0.04
BMI** (weight(kg)/height(m^2))				
Overweight	1.62(1.16 to 2.27)	0.005	2.07(1.04 to 4.09)	0.04
Obese	1.38(0.83 to 2.30)	0.2	2.41(1.05 to 5.54)	0.04
Waist circumference* (cm)				
Q2	1.63(1.05 to 2.53)	0.02	1.21(0.56 to 2.62)	0.6
Q3	1.79(1.16 to 2.76)	0.008	0.92(0.40 to 2.10)	0.8
Q4	1.57(0.98 to 2.50)	0.06	1.17(0.49 to 2.80)	0.7
Systolic BP* (mmHg)				
Q2	0.61(0.40 to 0.92)	0.01	0.51(0.24 to 1.09)	0.08
Q3	0.60(0.40 to 0.91)	0.01	0.69(0.35 to 1.38)	0.3
Q4	0.99(0.68 to 1.43)	0.9	0.36(0.15 to 0.84)	0.02
Diastolic BP* (mmHg)				
Q2	0.71(0.47 to 1.06)	0.1	0.59(0.29 to 1.19)	0.1
Q3	0.88(0.60 to 1.30)	0.5	0.36(0.15 to 0.86)	0.02
Q4	1.23(0.84 to 1.81)	0.3	0.80(0.40 to 1.58)	0.5
HDL cholesterol* (mg/dL)				
Q2	0.60(0.42 to 0.87)	0.007	0.68(0.34 to 1.36)	0.3
Q3	0.55(0.35 to 0.86)	0.009	0.60(0.30 to 1.23)	0.2
Q4	0.45(0.30 to 0.67)	<0.001	0.55(0.24 to 1.25)	0.2
Triglycerides* (mg/dL)				
Q2	1.57(1.03 to 2.39)	0.03	0.82(0.32 to 2.08)	0.7
Q3	2.07(1.39 to 3.08)	<0.001	2.08(1.03 to 4.20)	0.04
Q4	2.57(1.67 to 3.96)	<0.001	1.84(0.89 to 3.79)	0.09
Fasting glucose* (mg/dL)				
Q2	1.10(0.73 to 1.65)	0.6	0.72(0.36 to 1.43)	0.3
Q3	0.99(0.65 to 1.49)	0.9	0.67(0.31 to 1.42)	0.3
Q4	1.17(0.79 to 1.73)	0.4	0.70(0.33 to 1.47)	0.4
Diabetes prevalence (yes vs. no)	1.75 (1.20 to 2.57)	0.004	0.74(0.32 to 1.72)	0.5

*Sex specific quartiles of factors, OR demonstrate comparison to lowest quartile (Q1)
**BMI OR demonstrate comparison to participants in underweight/normal category
-Abbreviations: BMI: body mass index, BP: blood pressure, HDL: high density lipoprotein cholesterol.
-Conversion factors: HDL cholesterol mg/dL to mmol/L, x 0.02586; Triglycerides mg/dL to mmol/L, x 0.01129; Fasting glucose mg/dL to mmol/L, x 0.05551
